# Remote Testing Apps for Multiple Sclerosis Patients: Scoping Review of Published Articles and Systematic Search and Review of Public Smartphone Apps

**DOI:** 10.2196/37944

**Published:** 2023-02-06

**Authors:** Jacob B Michaud, Cameron Penny, Olivia Cull, Eric Hervet, Ludivine Chamard-Witkowski

**Affiliations:** 1 Department of Internal Medicine Dalhousie University Halifax, NS Canada; 2 Centre de Formation Médicale du Nouveau-Brunswick Moncton, NB Canada; 3 Faculty of Medicine Dalhousie University Halifax, NS Canada; 4 Department of Computer Science Université de Moncton Moncton, NB Canada; 5 Department of Neurology Dr.-Georges-L.-Dumont University Hospital Center Moncton, NB Canada

**Keywords:** multiple sclerosis, mobile application, mobile phone, app, mHealth, eHealth, digital health, telehealth

## Abstract

**Background:**

Many apps have been designed to remotely assess clinical status and monitor symptom evolution in persons with multiple sclerosis (MS). These may one day serve as an adjunct for in-person assessment of persons with MS, providing valuable insight into the disease course that is not well captured by cross-sectional snapshots obtained from clinic visits.

**Objective:**

This study sought to review the current literature surrounding apps used for remote monitoring of persons with MS.

**Methods:**

A scoping review of published articles was conducted to identify and evaluate the literature published regarding the use of apps for monitoring of persons with MS. PubMed/Medline, EMBASE, CINAHL, and Cochrane databases were searched from inception to January 2022. Cohort studies, feasibility studies, and randomized controlled trials were included in this review. All pediatric studies, single case studies, poster presentations, opinion pieces, and commentaries were excluded. Studies were assessed for risk of bias using the Scottish Intercollegiate Guidelines Network, when applicable. Key findings were grouped in categories (convergence to neurological exam, feasibility of implementation, impact of weather, and practice effect), and trends are presented. In a parallel systematic search, the Canadian Apple App Store and Google Play Store were searched to identify relevant apps that are available but have yet to be formally studied and published in peer-reviewed publications.

**Results:**

We included 18 articles and 18 apps. Although many MS-related apps exist, only 10 apps had published literature supporting their use. Convergence between app-based testing and the neurological exam was examined in 12 articles. Most app-based tests focused on physical disability and cognition, although other domains such as ambulation, balance, visual acuity, and fatigue were also evaluated. Overall, correlations between the app versions of standardized tests and their traditional counterparts were moderate to strong. Some novel app-based tests had a stronger correlation with clinician-derived outcomes than traditional testing. App-based testing correlated well with the Multiple Sclerosis Functional Composite but less so with the Expanded Disability Status Scale; the latter correlated to a greater extent with patient quality of life questionnaire scores.

**Conclusions:**

Although limited by a small number of included studies and study heterogeneity, the findings of this study suggest that app-based testing demonstrates adequate convergence to traditional in-person assessment and may be used as an adjunct to and perhaps in lieu of specific neurological exam metrics documented at clinic visits, particularly if the latter is not readily accessible for persons with MS.

## Introduction

Multiple sclerosis (MS) has a fluctuating clinical course punctuated by relapses, remissions, and progressive deterioration for many affected patients. As such, the neurologist requires an accurate representation of the symptomatology of the patient with MS in order to evaluate the efficacy of treatment [1].

Infrequent and intermittent monitoring as provided at office visits may not truly reflect the day-to-day functioning and quality of life of patients living with MS [2]. Persons with MS may also have recall bias when reporting symptoms to their neurologist [2]. Additionally, symptoms in MS can fluctuate depending on fatigue, mood, and weather; thus, the cross-sectional nature of the information obtained from an individual clinic visit may be of limited accuracy compared with trends in symptoms over time [3,4]. The need for at-home MS follow-up has been further emphasized by the current COVID-19 pandemic, in which many medical centers have implemented in-person patient visit limits to reduce the spread of the virus [5].

Remote evaluation of clinical status and symptoms in persons with MS could serve as a means of obtaining additional information that is not provided by the traditional office visit. Many apps for remote assessment of persons with MS exist, ranging from symptom logs, patient-reported outcome trackers, assessments of cognitive function and fine motor skills, as well as drug adherence and adverse drug event trackers [6-8]. The objective of this review was to identify and evaluate apps designed to enable remote assessment of persons with MS and whether the means of assessment utilized in these various apps are supported by scientific evidence.

## Methods

### Review Sources

A scoping review was performed to identify articles evaluating apps dedicated to the remote testing and follow-up of persons with MS. The PRISMA (Preferred Reporting Items for Systematic Reviews and Meta-Analyses) guidelines were followed for this portion of the review [9]. No protocol for this review was previously published.

A separate review of the Canadian Apple App Store and Google Play Store was conducted in parallel. This was done in order to identify apps available for public use, including some identified in the literature search as well as those that had not been formally studied prior to dissemination.

### Eligibility Criteria

Scientific papers were included if they met the following criteria: The study evaluated the use of remote monitoring of persons with MS via smartphone or tablet app and was published in English, French, or Spanish prior to January 17, 2022. Cohort studies, feasibility studies, and randomized controlled trials were included in this review. Studies were included if the application was used to measure one or more of the following functional domains: physical disability, fatigue, visual symptoms, urinary symptoms, balance, mood symptoms, pain, cognition, or ambulation. Exclusion criteria included pediatric studies, single case studies, poster presentations, opinion pieces, and commentaries.

Publicly available apps that were intended for symptom tracking or app-based testing of persons with MS were included in the app review portion of this paper if they were able to measure one or more of the aforementioned metrics.

### Search Strategy

**Figure 1:**
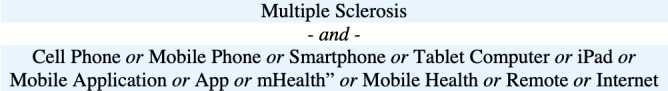
Search strategy.

PubMed/Medline, EMBASE, CINAHL, and Cochrane databases were searched from inception to January 17, 2022, to identify studies suitable for inclusion. The search strategy is detailed in Figure 1, and the detailed search strategy is presented in Multimedia Appendix 1.

As for the apps, the Canadian iOS Apple App Store and Android Google Play Store were searched using the term “Multiple Sclerosis” for publicly available apps.

### Data Collection and Analysis

Two authors (JBM and CP) independently screened studies for the inclusion criteria based on title and abstract. The articles were then subject to an independent full-text review, and inclusion was determined by consensus. The references of included studies were screened to identify any additional articles suitable for inclusion that were not captured in the initial search strategy. The aforementioned authors collected data on application testing metrics as well as on convergence with standard neurological exam findings (Pearson correlation coefficients and linear mixed effects estimates), feasibility of implementation (qualitative assessment and adherence rates), weather analysis, and practice effect. Data collection also included participant age, diagnosis, baseline Expanded Disability Status Scale (EDSS), study design, study funding, and follow-up period. Authors JBM and OC assessed included articles for risk of bias using the Scottish Intercollegiate Guidelines Network (SIGN) checklist, when applicable [10]. Relevant articles were grouped in primary outcome categories, and data were presented qualitatively.

Authors JBM and CP independently screened the title and description of the apps, and inclusion was determined by consensus. The included apps were then reviewed, and the functional domains evaluated were documented.

### Presentation

For the purpose of readability, this article considered correlation coefficients |*r*|≥0.75 to be strong, 0.75>|*r*|≥0.5 to be moderate, 0.5>|*r*|≥0.25 to be weak, and |*r*|<0.25 to not be correlated.

## Results

### Study and App Identification

**Figure 2:**
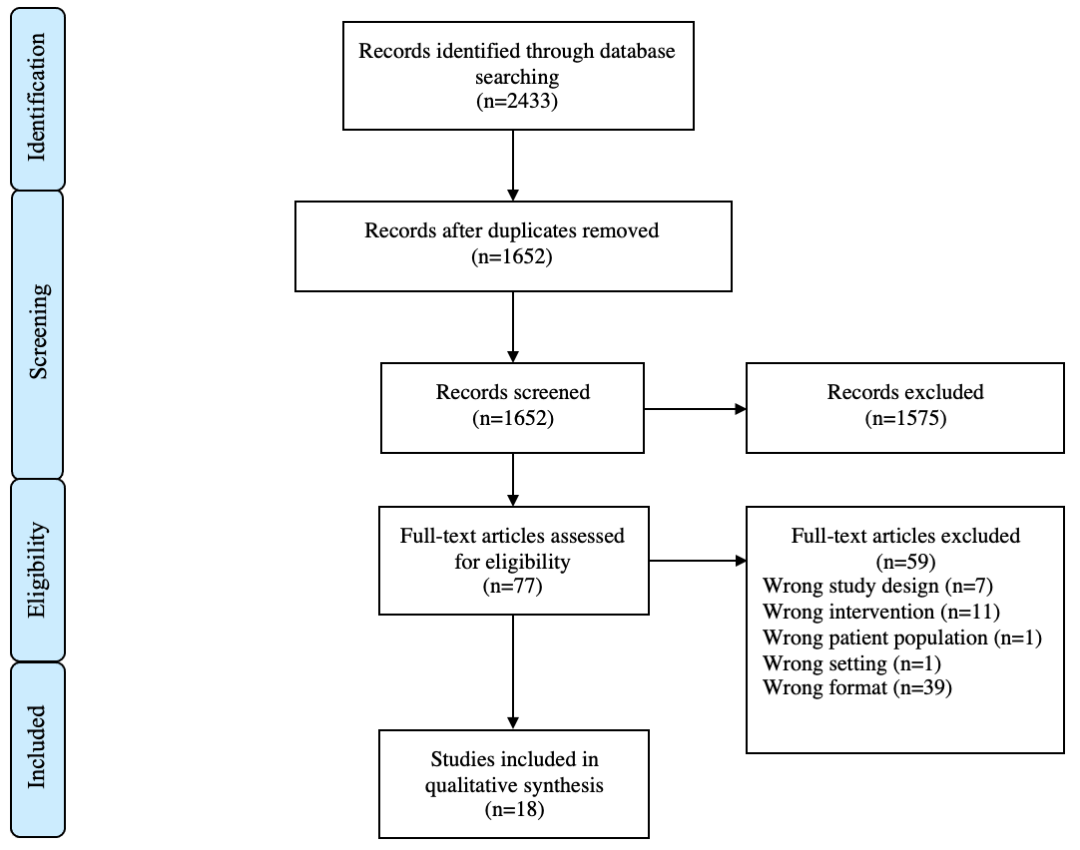
Included articles.

A total of 2433 studies were identified using the search strategy defined in the Methods section. Following duplicate removal and title and abstract screening, 77 studies were selected for full-text review. Of these studies, 18 were in keeping with the predefined inclusion criteria (Figure 2). All 18 studies were found to be of acceptable or high quality using the SIGN checklist [10].

**Figure 3:**
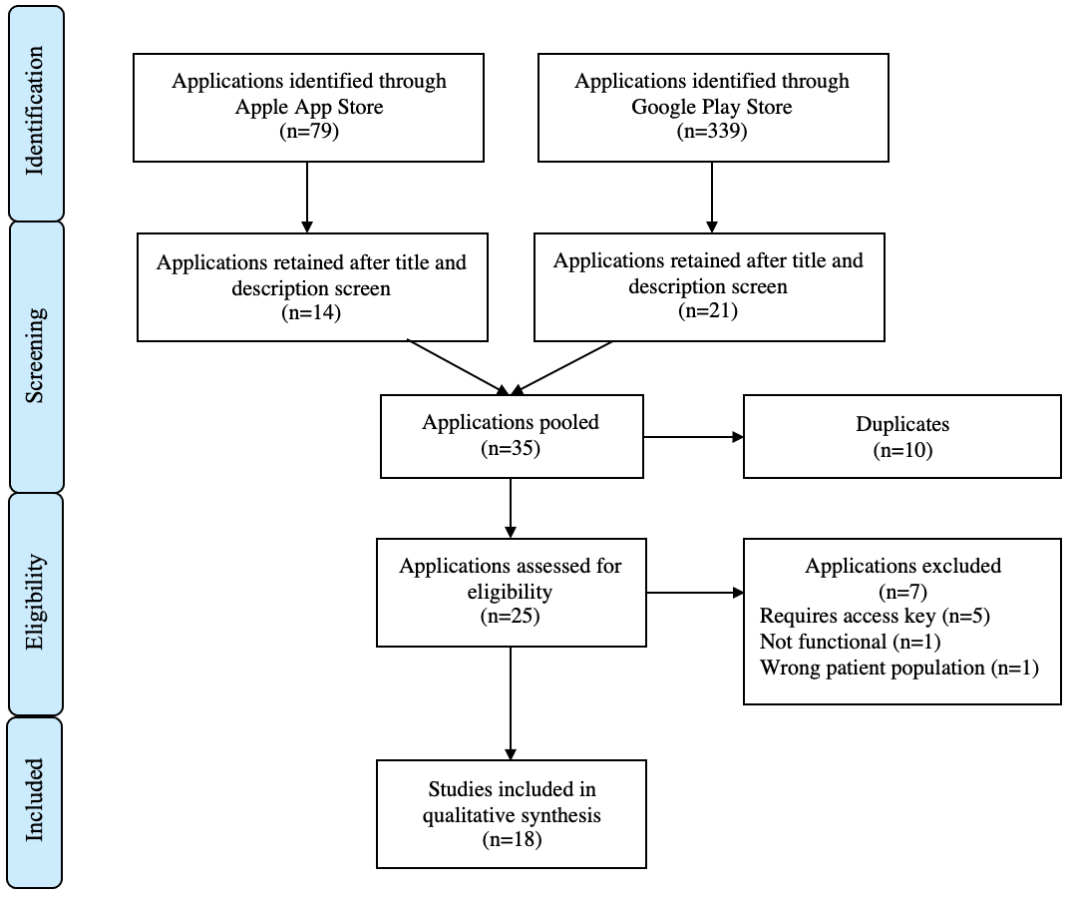
Included apps.

As for the app store review, the search yielded 79 apps in the Apple App Store and 339 apps in the Google Play Store. After removal of duplicates and title and description screening, 25 apps were selected for full app review. Of these apps, only 18 were deemed to fit the inclusion criteria (Figure 3). Of the 18 apps included, 2 had supporting literature that was identified in the scoping review portion of this paper [11-14].

### Characteristics of Included Studies

Of the 18 articles included, 12 sought to compare apps with a neurologist exam, disability scale, or recognized standardized tests [11-13,15-23]. The feasibility of implementing an app designed for remote monitoring of persons with MS was evaluated in 3 studies [24-26], 2 articles compared quality of life questionnaires with app-based functional tests and clinician-reported outcomes [25,27], and 2 apps assessed the local weather’s impact on persons with MS-reported fatigue and app functional test results [25,28]. Finally, 1 article evaluated the practice effect of repeated at-home MS testing [14] (Table 1).

**Table 1:** Characteristics of included studies.

Author(s), year	Countries	Study design	Type of multiple sclerosis
Hsu et al, 2021 [22]	United States	Prospective cohort	RR^a^, PP^b^, SP^c^, CIS^d^, unknown
Golan et al, 2021 [27]	Israel	Prospective cohort	RR, PP, SP
Pratap et al, 2020 [28]	United States	Prospective cohort	RR, PP, SP
Hsu et al, 2021 [15]	United States	Cross-sectional	RR, PP, SP, CIS
Newland et al, 2019 [26]	United States	Descriptive study	RR, SP
Midaglia et al, 2019 [24]	Spain, United States	Prospective cohort	RR, PP, SP
Montalban et al, 2021 [12]	Spain, United States	Prospective cohort	RR, PP, SP
Woelfle et al, 2021 [14]	Switzerland	Prospective cohort	N/A^e^
Lam et al, 2021 [18]	Netherlands	Prospective cohort	RR, PP, SP
van Oirschot et al, 2020 [19]	Netherlands	Prospective cohort	RR
van Oirschot et al, 2021 [23]	Netherlands	Prospective cohort	RR
Boukhvalova et al, 2018 [16]	United States	Cross-sectional	RR, PP, SP
Boukhavalova et al, 2019 [17]	United States	Prospective cohort	RR, PP, SP
Maillart et al, 2019 [11]	France	Crossover study	RR, PP
Tanoh et al, 2021 [13]	France	Prospective cohort	RR, PP
Bove et al, 2015 [25]	United States	Prospective cohort	RR, PP, SP, CIS
Lam et al, 2021 [20]	Netherlands	Prospective cohort	RR, PP, SP
Lam et al, 2022 [21]	Netherlands	Prospective cohort	RR, PP, SP

^a^RR: relapsing remitting multiple sclerosis.

^b^PP: primary progressive multiple sclerosis.

^c^SP: secondary progressive multiple sclerosis.

^d^CIS: clinically isolated syndrome.

^e^N/A: not available.

### Characteristics of Included Apps

Of the 18 apps included, 5 had objective symptom testing through mobile phone sensors. The other 13 did not have active tests but did allow for symptom logging. Of the apps included in this study, 2 had complimentary data that were identified during the scoping review portion of the current study.

Four apps were exclusively found on the Apple App Store, 8 apps were exclusively found in the Google Play Store, and 6 apps were found in both stores. All but 2 of the apps included were free of charge.

### Scoping Review Outcomes

As aforementioned, the reviewed articles were categorized according to 4 main objectives: evaluating convergence with neurological exam, feasibility of implementation of an app for persons with MS, evaluating the practice effect of repeated at-home testing, and comparing app-based tests with quality of life questionnaires and local weather.

#### Convergence With the Neurological Exam

Of the 18 articles, 14 articles described 12 apps that measured physical disability and correlated these with findings on clinical exam. These measures of physical disability were done by tap tests [16], shape drawing tests [11,13], pinching tests [12], assessment of passively acquired keyboard metrics [18,20], or using a level test wherein one must balance their phone in order to keep a ball in a designated screen area [17]. Visual symptoms were measured in 2 apps using tests of steering around obstacles [15] or a mobile vision test [11]. Cognitive function was measured in 6 apps: 3 apps used an electronic version of the Symbol Digit Modalities Test (SDMT) [11-13,18,19]; 1 used a go-no go test coupled with multitasking and visuomotor steering [15]; 1 used a battery of attention, working memory, and goal management evaluations [22]; and 1 measured keystroke dynamics including keystroke latency, emoji use, and word length [20,21]. Ambulation was measured in 3 apps using an app-based timed 25-foot walk test (T25FW) [11,13], 2-minute walk test (2MWT) [23], U-turn test [12], or maximum distance walked test [11,13]. The main tests and functional domains can be found in Table 2.

One study compared the MS Suite app balloon popping test to the 9-Hole Peg Test (9HPT) and found that the app slightly outperformed the 9HPT in its ability to correlate with clinician-derived outcomes [16]. The number of balloons popped correlated strongly with cerebellar function and moderately with upper extremity strength and motor exam. The study also included 4 patients who could no longer perform the 9HPT due to severe disease but were able to perform the balloon popping test.

**Table 2:** App tests from scientific articles and comparators for convergence with neurological exam or patient questionnaires.

App and functional domains		App test	Comparator
**Adaptive Cognitive Evaluation** [22]			
	Cognition	Boxed task, sustained attention task, spatial span	SDMT^a^
**ElevateMS** [28]			
	PD^b^	Finger tapping, finger to nose	PDDS^c^, Neuro-QoL^d^
	Ambulation, balance	Walk and balance test	PDDS, Neuro-QoL
	Cognition	Voice-controlled DSST^e^	PDDS, Neuro-QoL
**Evo Monitor** [15]			
	PD	Go/no go, tilt to steer, and combination of both tasks	MSFC-4^f^, EDSS^g^
	Cognition	Go/no go, tilt to steer, and combination of both tasks	BICAMS^h^
**Floodlight** [12,14]			
	PD	Draw a shape, pinching test	9HPT^i^, EDSS
	Balance	Static balance test	BBS^j^
	Cognition	sSDMT^k^	SDMT
	Ambulation	2MWT^l^, U-turn test	T25FW^m^, EDSS
**MSCopilot** [11,13]			
	PD	Spiral test	9HPT
	Visual	Vision test	SLCLAT^n^
	Cognition	Cognition test (sSDMT)	SDMT, PASAT^o^
	Ambulation	Walking test	T25FW, EDSS
**MS Sherpa** [18,19,23]			
	Cognition	sSDMT	SDMT
	Ambulation	e-2MWT^p^	2MWT
**MS Suite** [16,17]			
	PD	Balloon popping, tap test, tilt test	NeurEx^q^, EDSS
	Cognition	Tilt test	SDMT
**NeuroKeys** [20,21]			
	PD	Press-press latency, release-release latency, hold time, flight time, precorrection slowing, correction duration, post correction slowing, after punctuation pause, emoji sentiment score [11]	EDSS, 9HPT
	Cognition	Press-press latency, release-release latency, hold time, flight time, precorrection slowing, correction duration, post correction slowing, after punctuation pause, emoji sentiment score [11]	SDMT
	Fatigue	Press-press latency, release-release latency, hold time, flight time, precorrection slowing, correction duration, post correction slowing, after punctuation pause, emoji sentiment score [11]	CIS-F^r^

^a^SDMT: Symbol Digit Modalities Test.

^b^PD: physical disability.

^c^PDSS: Patient-Determined Disease Steps.

^d^Neuro-QoL: Quality of Life in Neurological Disorders.

^e^DSST: Digit Symbol Substitution Test.

^f^MSFC-4: Multiple Sclerosis Functional Composite 4.

^g^EDSS: Expanded Disability Status Scale.

^h^BICAMS: Brief International Cognitive Assessment for Multiple Sclerosis.

^i^9HPT: 9-Hole Peg Test.

^j^BBS: Berg Balance Scale.

^k^sSDMT: smartphone SDMT.

^l^2MWT: 2-minute walk test.

^m^T25FW: timed 25-foot walking test.

^n^SLCLAT: Sloan Low Contrast Letter Acuity Test.

^o^PASAT: Paced Auditory Serial Addition Test.

^p^e-2MWT: electronic 2MWT.

^q^NeurEx: digitalized neurological examination.

^r^CIS-F: Checklist Individual Strength Fatigue subscale.

Keystroke dynamics were found to have weak correlation with the EDSS and moderate correlation with the SDMT in 1 study [20]. Another found that the use of emojis with more neutral sentiment as well as decreased word length were responsive to changes in the EDSS in a manner that was statistically significant [21].

One study evaluating the correlation of the smartphone SDMT (sSDMT) with the traditional SDMT found a moderate correlation for tests done in the morning and in the evening for the MS Sherpa app [18]. In 2 other studies comparing MS Sherpa’s sSDMT as well as Floodlight’s sSDMT to the traditional SDMT, strong correlations were found between these tests [12,19].

Two studies compared their app-based tests with the SDMT. The first compared the Evo Monitor multitasking test with SDMT and found a moderate correlation [15]. The second compared the SDMT and MS Suite level test, in which the time a virtual ball stayed in the center of the screen was found to correlate moderately with the SDMT [17]. These same 2 studies compared the multitasking test and level test with the EDSS. Both correlated weakly with the EDSS [15,17].

The MS Copilot app included several tests: spiral drawing test, maximum distance walked without stopping, verbal SDMT, and low contrast vision test. The *z* score of participants’ test batteries correlated strongly with the Multiple Sclerosis Functional Composite (MSFC) *z* score [11]. Another MS Copilot battery comprising of maximum walking distance, shape drawing, and SDMT correlated moderately with the EDSS [13].

In 1 study, the Floodlight app’s pinching test correlated moderately with the 9-HPT. It also found that Floodlight’s U-turn test correlated moderately with the T25FW. Of the Floodlight tests, the U-turn test had the strongest correlation with the EDSS despite the weak correlation (*r*=–0.45; *P*<.001) [12]. Individual test scores were not compounded in this study as they were in the MS Copilot study [13].

Finally, MS Sherpa’s smartphone 2MWT measurements were found to be approximately 8.43 meters greater than those measured traditionally. In this same study, there was no statistically significant correlation identified between the app-based 2MWT and EDSS [23].

#### Feasibility of Implementation

The feasibility of implementing an app to monitor symptoms in persons with MS was assessed in 3 studies. Adherence rates were 51% for an app requiring 12 months of daily data collection (n=38) [25]; 70% for an app requiring daily, weekly, fortnightly, or on-demand activities (n=76) [24]; and 87% for an app requiring 7 consecutive days of testing and a repeat test 4 weeks later (n=32) [26].

#### Quality of Life Questionnaires

App-based quality of life questionnaires were evaluated in 2 studies: 1 compared app-derived neurological quality of life (Neuro-QoL) questionnaires to in-app functional tests. Using a linear mixed effects model, the study found that the following Neuro-QoL domains correlated significantly with app tests: Upper extremity function was correlated with finger tapping test, lower extremity function was correlated with walk and balance tests, and cognitive function was correlated with the voice-based Digit Symbol Substitution Test (DSST) [28].

Another study assessed the e-Diary app, in which an app-based questionnaire was used to derive a Bodily Function Summary Score that was then compared to clinician-reported outcomes. This study found a strong correlation between the Bodily Function Summary Score and EDSS scores [27].

#### Weather

Whether increasing local temperature had a negative impact on in-app tests was evaluated in 2 studies [25,28]. The first included 495 persons with MS and found that increasing temperature had a significant negative impact on finger tapping, DSST, and finger to nose [28]. However, the second study, following 22 persons with MS, found no statistically significant association between the Modified Fatigue Inventory Scale and temperature or daylight hours [25].

#### Practice Effect

The development of a practice effect with repeated at-home app-based MS testing was assessed in 1 study. Data included in this study were derived from the Floodlight app. Domains assessed included daily repetition of finger pinching, shape drawing, 2MWT, U-turn test, static balance test, and weekly repetition of virtual SDMT. The study found improvement in test scores ranging from 11% to 54.2% on daily repetition of tests with the exception of the 2MWT. For the sSDMT, an average improvement of 40.8% was observed after 5 weeks of weekly testing [14].

The key findings of each included article are presented in Table 3.

**Table 3:** Key findings of included studies.

App and author, year		All assessed functional domains	Number of people with MS^a^	Key findings
**Adaptive Cognitive Evaluation**				
	Hsu et al, 2021 [22]	Cognition	53	Boxed reaction time of persons with MS correlated most strongly with SDMT^b^ (*r*=–0.50; *P*<.001), including when covariates were accounted for (*r*=–0.43; *P*=.002). Sustained attention span and spatial span were not significantly correlated with SDMT.
**e-Diary**				
	Golan et al, 2021 [27]	PD^c^, visual, urinary, mood, pain, cognition	97	e-diary–derived PROs^d^ were significantly correlated with corresponding functional system scores (0.38<*r*<0.8; *P*<.001). The sum of bodily functions showed a correlation coefficient of *r*=0.77 (*P*<.001) with clinician EDSS^e^.
**ElevateMS**				
	Pratap et al, 2020 [28]	PD, balance, cognition, weather	495	Neuro-QoL^f^ categories correlated significantly with finger tapping (β^g^=0.4; *P*<.001), walk and balance (β=–99.18; *P*=.02), and DSST^h^ (β=1.60; *P*=.03). Baseline PDDS was associated with finger to nose (β=.01; *P*=.01). Increasing temperature had a significant impact on finger tapping, DSST (β=–.06; *P*=.009), and finger to nose.
**Evo Monitor**				
	Hsu et al, 2021 [15]	PD, cognition	100	Evo Monitor multitasking test was associated with SDMT (*r*=0.52; *P*<.001), EDSS (*r*=–0.35; *P*<.01), and T25FW^i^ (*r*=–0.41; *P*<.001). Go/no go and tilt to steer tests were associated with SDMT (*r*=–0.31; *P*=.001 and *r*=0.40; *P*<.001, respectively).
**Fatigue**				
	Newland et al, 2019 [26]	PD, pain, cognition	32	Most participants (87%) completed all of the surveys required (7 consecutive days and repeat 4 weeks later).
**Floodlight**				
	Midaglia et al, 2019 [24]	PD, fatigue, balance, mood, pain, cognition, ambulation	76	70% of participants were adherent to all active tests. 75% of participants were adherent to all tests except 2MWT^j^. Mean satisfaction with the app at week 12 was 74.1% and at week 24 was 73.7%.
	Montalban et al, 2021 [12]	PD, balance, cognition	76	Strongest correlation was found between sSDMT^k^ and SDMT (*r*=0.82, *P*<001). Pinching test correlated with 9HPT^l^ (*r*=0.64, *P*<.001). U-turn test correlated with T25FW (*r*=–0.52, *P*<.001). Strongest correlation with EDSS was with U-turn test (*r*=–0.45, *P*<.001). Static balance test was not significantly associated with Berg Balance Scale.
	Woelfle et al, 2021 [14]	PD, balance, cognition, ambulation	171-262	sSDMT, when repeated at 7-day intervals, had an average improvement of 40.8%. The practice effect was reached after 11 repetitions for one-half and after 35 repetitions for 90%. Finger pinching, draw a shape, U-turn, and static balance had average improvements of 54.2%, 23.9%, 11.0%, and 28.6%, respectively. 2MWT was not significantly associated with improvement.
**MS Copilot**				
	Maillart et al, 2019 [11]	PD, visual, cognition, ambulation	141	App combined task *z* score correlated with the MSFC^m^ *z* score (*r*=0.81; *P*<.001).
	Tanoh et al, 2021 [13]	PD, visual, cognition, ambulation	116	Summed scores of maximum walking distance, draw a shape, and mobile SDMT correlated with EDSS (*r*=–0.65; *P*<.001).
**MS Sherpa**				
	Lam et al, 2021 [18]	Cognition	102	sSDMT and SDMT correlation coefficients were *r*=0.687 (*P*<.001) in the morning and *r*=0.622 (*P*<.001) in the evening, with a regression coefficient of 0.87.
	van Oirschot et al, 2020 [19]	Cognition	25	The interclass correlation coefficient between SDMT and sSDMT results was 0.784, and the Pearson correlation coefficient was *r*=0.85 (*P*<.001).
	van Oirschot et al, 2021 [23]	Cognition, ambulation	25	Distance walked on e-2MWT was, on average, 8.43 meters greater than that with traditional 2MWT. There was no significant correlation between EDSS and e-2MWT.
**MS Suite**				
	Boukhvalova et al, 2018 [16]	PD, cognition	76	Balloon popping had correlation coefficients of *r*=0.62, *r*=0.75, and *r*=0.62 (*P*<.0001) with upper extremity strength, cerebellar function, and upper extremity motor exam, respectively. These values were *r*=0.59, *r*=0.57, and *r*=0.61 for the traditional 9HPT. Tap test was associated with 9HPT (*r*=0.66; *P*<.0001)
	Boukhvalova et al, 2019 [17]	PD, cognition	112	Level test time spent in center of the level test correlated with SDMT (*r*=0.57; *P*<.0001) and, to a lesser degree, with EDSS (*r*=–0.35; *P*<.01).
**N/A^n^**				
	Bove et al, 2015 [25]	PD, balance, cognition, weather	38	Adherence rate for the app was 51% at 12 months. Of those who completed the 1-year study (n=22), no significant association between MFIS^o^ and temperature (*P*=.18) nor daylight hours (*P*=.091) was noted.
**Neuro keys**				
	Lam et al, 2021 [20]	PD, cognition, fatigue	85	EDSS was most correlated with latency between key release (*r*=0.407, *P*<.001). Overall, the release-release latency keystroke metric correlated the most with SDMT (*r*=–0.553 *P*<.01).
	Lam et al, 2022 [21]	PD, cognition	94	The keystroke features most responsive to changes in EDSS were emoji sentiment neutrality and word length, with AUCs^p^ of 0.79 and 0.72, respectively.

^a^MS: multiple sclerosis.

^b^SDMT: Symbol Digit Modalities Test.

^c^PD: physical disability.

^d^PROs: patient-reported outcomes.

^e^EDSS: Expanded Disability Status Scale.

^g^Neuro-QoL: quality of life in neurological disorders.

^g^Linear mixed effects estimate.

^h^DSST: Digit Symbol Substitution Test.

^i^T25FW: timed 25-foot walk.

^j^2MWT: 2-minute walk test.

^k^sSDMT: smartphone SDMT.

^l^9HPT: 9-Hole Peg Test.

^m^MSFC: Multiple Sclerosis Functional Composite.

^n^N/A: not available.

^o^MFIS: Modified Fatigue Impact Scale.

^p^AUCs: areas under the curves.

### App Review

Of the 18 identified apps, 5 had a remote testing function. Of the 5 apps with remote testing abilities, all tested for physical disability and fine motor skills. Assessment of motor skills was done through tapping tests as in BeCare and MS Care Connect; drawing a shape or following a path as in Floodlight, MS Care, and MS Copilot; or a 9HPT equivalent as in Neurons. With regard to disability, 1 app, BeCare, measured arm raises, while Floodlight measured pinch and thumb strength.

Visual symptoms were evaluated by 3 of the apps. This was done by contrast sensitivity tests and measured optokinetic nystagmus as in BeCare, color vision tests as in MS Care Connect, or low-contrast vision tests as in MS Copilot.

Cognitive testing was performed in all 5 apps: 4 apps (BeCare, Floodlight, MS Care Connect, and MS Copilot) used the SDMT; 2 apps used modified versions of recognized MS tests like the Paced Auditory and Visual Serial Addition Test as in Neurons and the Stroop test as in BeCare; and some apps used other tests like stacking donuts in ascending size on pegs, memorizing words and matching them to categories, and tapping blocks in a memorized sequence as in MS Care Connect or memorizing animals as in BeCare.

All 5 apps had measures of ambulation: 3 apps (BeCare, Neurons, and MS Care Connect) had the T25FW, and 2 apps had time-limited walk tests such as BeCare’s 6-minute walk test or Floodlight’s 2MWT. BeCare also measured the Timed Up and Go test. Floodlight implemented passive monitoring of daily ambulation, while MS Copilot measured maximum distance walked.

Only 1 app, Floodlight, had a dedicated static balance test. Another app, MS Care Connect, measured reaction time. The BeCare app measured the ability to discriminate between mobile device vibration frequency. That same app also had an audio transcription test.

Symptom logging functions were found in 13 other apps, either through free-text entry or selecting within a list of suggested neurological symptoms. These are included in Table 4.

**Table 4:** Characteristics of included apps.

App name	Platform	Developer	Brief description
Aby	Both	Biogen Inc	Log MS^a^ symptoms
Bearable - Symptom and Mood Tracker	GPS^b^	Bearable	Log MS symptoms
BeCare MS Link	Both	BeCare Link LLC	Testing for PD^c^, visual, cognitive, ambulation, mood
Emilyn: My MS Companion	Both	BreakthroughX Health GmbH	Log MS symptoms
Floodlight^d^	Both	Roche SAS	Log MS symptoms; testing for PD, cognitive, balance, ambulation
Healthstories MS	AAS^e^	Jacob Wachsman	Log MS symptoms
icompanion	Both	Icometrix Inc	Log MS symptoms, may perform prEDSS^f^ or Neuro-QoL^g^
Innov SEP	GPS	Mallouki Adil	Log MS symptoms.
MSAA-My MS Manager	AAS	At Point of Care, LLC	Log MS symptoms, generate MFIS^h^ score
MS Care Connect	GPS	InterPro Bioscience Inc	Log MS symptoms; testing for PD, cognitive, ambulation
MSCopilot^d^	GPS	Ad Scientiam	Testing for PD, visual, cognitive, ambulation
MS Corner	GPS	Progentec Diagnostics	Log MS symptoms
MS Notes Journal	GPS	Roger Hartley	Log MS symptoms
MS Relapse Tool	Both	Darin Okuda	Log MS symptoms
MS Relapse Tracker/MS Attack	AAS	Flavia Chapa	Log MS symptoms, relapse probability assessment
Multiple Sclerosis Manager	GPS^f^	KingFishApps	Log MS symptoms.
Multiple Sclerosis Messenger	GPS	KingFishApps	Log MS symptoms and may send to MS nurse
Neurons	AAS	shazino	Testing for PD, cognitive, ambulation

^a^MS: multiple sclerosis.

^b^GPS: Google Play Store.

^c^PD: physical disability.

^d^App found to have supporting literature in the scoping review of scientific evidence.

^e^AAS: Apple App Store.

^f^prEDSS: patient-reported Expanded Disability Status Scale.

^g^Neuro-QoL: quality of life in neurological disorders.

^h^MFIS: Modified Fatigue Impact Score.

## Discussion

This review sought to evaluate and summarize available literature and apps assessing remote testing for persons with MS. Though well-designed studies evaluating concordance between app testing and the neurological exams do exist, many apps operate outside the realm of currently available scientific evidence.

### Comparison With Prior Work

To the authors’ knowledge, this is the first scoping review with a specific focus on the use of apps for symptom monitoring and tracking clinical course in persons with MS. Previous reviews on this topic have employed a wider scope, examining all clinical trials with data pertaining to apps used in MS [6,7], while others narrowed the scope to apps used for self-assessment and rehabilitation [29] or to gait and postural control [30]. Of the 2 reviews with wider scopes, one was published in 2018 and predates all but one of the included articles [6], and the other included only 3 studies that focused on apps employing dexterity tests, accelerometers, or other sensing technologies [7].

### Principal Findings

Many of the included studies demonstrated concordance between mobile testing for MS and various aspects of the neurological exam [11-13,15-23]. For example, the Adaptive Cognitive Evaluation, Elevate MS, EVO monitoring, Floodlight, MS Copilot, MS Suite, and NeuroKeys have all shown statistically significant correlations between the app and the physician’s exam. The strongest correlation coefficients with standardized scales were seen with MS Copilot, when test results were pooled and compared with the MSFC [11]. However, pooled results did not have the same correlation strength with the EDSS. This may reflect the stronger similarities in the MS Copilot battery and the tests administered during the MSFC.

Although the EDSS remains an important aspect of the evaluation of persons with MS both in clinic and in the context of clinical trials, most apps seeking to correlate in-app testing with EDSS have obtained weak to moderate, albeit statistically significant, correlation coefficients [12,13,15,18,20]. The correlation coefficients were much greater with app-based e-diary scores [27]. This is notable, as the EDSS has previously been criticized for its poor assessments of upper limb and cognitive functions, which are 2 domains that are evaluated in most apps for which published data exists [31]. Additionally, the EDSS’s nonlinearity may make it more difficult for testing-based apps to correctly obtain the EDSS score based on quantitative data derived from app-based testing [32].

One advantage to app-based evaluation of persons with MS is that virtual tests can be performed by persons with MS with more significant disability. One study found that some persons with MS were unable to perform the 9HPT yet were able to participate in app-based testing [16]. That said, app-based testing may be an obstacle to those with MS-related visual impairment who rely on tactile sensations to complete the required testing.

In terms of feasibility, adherence rates to the apps were lower for apps requiring daily participation for extended periods and higher for apps with less frequent testing [24-26]. This would suggest that adherence would be higher for apps that require less frequent active participation from persons with MS. Thus, striking the optimal balance between participant engagement and the adequacy of remote monitoring becomes important.

The increased frequency of app-based testing, when compared with infrequent office testing, may improve certain test results due to repeated practice. Woelfle et al [14] demonstrated improvement related to practice effect in most of the tests that comprise the Floodlight testing battery, an app that allows users to perform tests daily or weekly; however, this practice effect was not observed with the 2MWT, which evaluates walking, an activity generally performed daily by those who remain ambulatory. Similar practice effects have been described for the MSFC [33]. Clinicians who plan to use app-based testing as part of their evaluation of persons with MS should be wary of these effects when interpreting results, as they may mask deterioration or feign clinical improvement. Where applicable, a possible mitigation strategy would be to use alternating versions of tests. No studies have yet determined the optimal testing interval to avoid practice effect–related improvement.

Data on local temperature and its impact on app-based test performance have shown that increasing temperatures correlate negatively with test scores [28]. As such, apps that monitor local temperature may offer additional insight to the MS specialist who may not consider this factor when evaluating persons with MS.

Although many apps designed to track symptoms in persons with MS are publicly available on app stores, only 10 apps were identified in our scoping review as having published evidence supporting their use.

### Limitations

This scoping review is limited first by the relatively small number of included articles as well as the heterogeneity of included articles. This renders drawing generalized conclusions difficult given the limited number of studies and the different comparators. As more data become available with the growth of mobile health (mHealth), future reviews may be able to compare different testing metrics with more certainty. The second limitation relates to the rapid evolution of mHealth publications and app development. This is supported by the fact that two-thirds of the included articles were published within the last 2 years. At the time of its publication, this review may not reflect the most recent data available.

### Future Directions

Future app developers may wish to include both objective measures of clinical status as well as patient-reported outcomes in order to aid the neurologist in evaluating persons with MS, especially if the app is to assess the EDSS. The mobile version of the SDMT correlated well with the traditional SDMT and could be included as a measure of cognitive decline. Although current research does not describe the optimal testing interval, app testing should be used sparingly to encourage participation and reduce the practice effect. Developers may also wish to include local weather data at time of testing to allow for contextualization of at-home results.

### Conclusion

The current review serves as a summary of the existing apps designed for monitoring of persons with MS and their supporting literature. Current evidence demonstrates adequate convergence of app-based testing to traditional in-person assessment. Although persons with MS will likely always require the human interaction of in-person follow-up, apps may be used as an adjunct to these visits for patients who are unable to see their neurologist on a regular basis. Although many apps with remote testing abilities are available to the public, a minority have published evidence supporting their use. Several apps had unique beneficial features; however, there was a significant amount of redundancy. Most app-based tests had a focus on physical disability and cognition. There remains a need for a comprehensive validated app that combines both patient-reported outcomes and multiple types of remote testing to better understand and care for persons with MS.

## Appendix

Multimedia Appendix 1 Detailed search strategy.

Multimedia Appendix 2 Data sets.

## Abbreviations

2MWT: 2-minute walk test
9HPT: 9-Hole Peg Test
DSST: Digit Symbol Substitution Test
EDSS: Expanded Disability Status Scale
mHealth: mobile health
MS: multiple sclerosis
MSFC: Multiple Sclerosis Functional Composite
Neuro-QoL: neurology quality of life
SDMT: Symbol Digit Modalities Test
SIGN: Scottish Intercollegiate Guidelines Network
sSDMT: smartphone SDMT
T25FW: timed 25-foot walk test

## Data Availability

The data sets generated during or analyzed during the current study are available in Multimedia Appendix 2.
