# Supplementary material for: Remote Testing Apps for Multiple Sclerosis Patients: Scoping Review of Published Articles and Systematic Search and Review of Public Smartphone Apps
Source: JMIR Neurotechnol. 2023 Feb 6;2:e37944. doi: 10.2196/37944 (PMC12671287; doi:10.2196/37944)
Supplement: Multimedia Appendix 1 [file neuro_v2i1e37944_app1.docx]

**Multimedia Appendix 1.** Detailed search strategy.

All searches were run on January 17, 2022.

## PubMed/MEDLINE

| 3,"(""Multiple Sclerosis""[Title/Abstract]) AND (((((((((((""Cell Phone""[Title/Abstract]) OR (""Mobile Phone""[Title/Abstract])) OR (""Smartphone""[Title/Abstract])) OR (""Tablet Computer""[Title/Abstract])) OR (""iPad""[Title/Abstract])) OR (""Mobile Application""[Title/Abstract])) OR (""App""[Title/Abstract])) OR (""mHealth""[Title/Abstract])) OR (""Mobile Health""[Title/Abstract])) OR (""Remote""[Title/Abstract])) OR (""Internet""[Title/Abstract]))",,,"""Multiple Sclerosis""[Title/Abstract] AND (""Cell Phone""[Title/Abstract] OR ""Mobile Phone""[Title/Abstract] OR ""Smartphone""[Title/Abstract] OR ""Tablet Computer""[Title/Abstract] OR ""iPad""[Title/Abstract] OR ""Mobile Application""[Title/Abstract] OR ""App""[Title/Abstract] OR ""mHealth""[Title/Abstract] OR ""Mobile Health""[Title/Abstract] OR ""Remote""[Title/Abstract] OR ""Internet""[Title/Abstract])" |
| --- |
| 2,"((((((((((""Cell Phone""[Title/Abstract]) OR (""Mobile Phone""[Title/Abstract])) OR (""Smartphone""[Title/Abstract])) OR (""Tablet Computer""[Title/Abstract])) OR (""iPad""[Title/Abstract])) OR (""Mobile Application""[Title/Abstract])) OR (""App""[Title/Abstract])) OR (""mHealth""[Title/Abstract])) OR (""Mobile Health""[Title/Abstract])) OR (""Remote""[Title/Abstract])) OR (""Internet""[Title/Abstract])",,,"""Cell Phone""[Title/Abstract] OR ""Mobile Phone""[Title/Abstract] OR ""Smartphone""[Title/Abstract] OR ""Tablet Computer""[Title/Abstract] OR ""iPad""[Title/Abstract] OR ""Mobile Application""[Title/Abstract] OR ""App""[Title/Abstract] OR ""mHealth""[Title/Abstract] OR ""Mobile Health""[Title/Abstract] OR ""Remote""[Title/Abstract] OR ""Internet""[Title/Abstract]" |
| 1,"""Multiple Sclerosis""[Title/Abstract]",,,"""Multiple Sclerosis""[Title/Abstract]" |

## EMBASE

#3

#1 AND #2

#2

'multiple sclerosis':ti,ab,kw

#1

'cell phone':ti,ab,kw OR 'mobile phone':ti,ab,kw OR smartphone:ti,ab,kw OR 'tablet computer':ti,ab,kw OR ipad:ti,ab,kw OR 'mobile application':ti,ab,kw OR app:ti,ab,kw OR mhealth:ti,ab,kw OR 'mobile health':ti,ab,kw OR remote:ti,ab,kw OR internet:ti,ab,kw

## CINAHL

S3 S1 AND S2 Expanders - Apply related words; Apply equivalent subjects

Search modes - Boolean/Phrase Interface - EBSCOhost Research Databases

Search Screen - Advanced Search

Database - CINAHL with Full Text

S2 AB multiple sclerosis

Expanders - Apply related words; Apply equivalent subjects

Search modes - Boolean/Phrase Interface - EBSCOhost Research Databases

Search Screen - Advanced Search

Database - CINAHL with Full Text

S1 AB Cell phone OR AB smartphone OR AB mobile phone OR AB tablet computer OR AB ipad OR AB mobile applications OR AB mobile application OR AB app OR AB mhealth OR AB mobile health OR AB remote OR AB internet Expanders - Apply related words; Apply equivalent subjects

Search modes - Boolean/Phrase Interface - EBSCOhost Research Databases

Search Screen - Advanced Search

Database - CINAHL with Full Text

## COCHRANE

ID Search

#1 (cell phone):ti,ab,kw OR (mobile phone):ti,ab,kw OR (smartphone):ti,ab,kw OR (tablet computer):ti,ab,kw OR (ipad):ti,ab,kw (Word variations have been searched)

#2 (mobile application):ti,ab,kw OR (app):ti,ab,kw OR (mhealth):ti,ab,kw OR (mobile health):ti,ab,kw OR (remote):ti,ab,kw (Word variations have been searched)

#3 (multiple sclerosis):ti,ab,kw (Word variations have been searched)

#4 (internet):ti,ab,kw (Word variations have been searched)

#5 #1 OR #2 OR #4

#6 #5 AND #3
